# Internal Benchmarking of Semi-Empirical Methods: Bromine-Containing Crystals as a Sensitive Test Case

**DOI:** 10.3390/molecules31081288

**Published:** 2026-04-15

**Authors:** Ilona A. Isupova, Denis A. Rychkov

**Affiliations:** 1 Institute of Solid State Chemistry and Mechanochemistry, Siberian Branch of the Russian Academy of Sciences, 18 Kutateladze Street, 630128 Novosibirsk, Russia; 2 Synchrotron Radiation Facility—Siberian Circular Photon Source “SKlF”, Boreskov Institute of Catalysis of Siberian Branch of the Russian Academy of Sciences (SRF “SKIF”), 1 Nikolsky Avenue, Koltsovo, 630559 Novosibirsk, Russia

**Keywords:** computational chemistry, density functional theory, DFTB, CrystalExplorer, benchmark, organic crystals, bromine

## Abstract

Selecting appropriate computational methods for organic crystals becomes particularly challenging for systems containing heavy halogens like bromine, whose complex electronic structures and diverse non-covalent interactions challenge approximate methods. Here we benchmark periodic DFT (PBE-D3BJ), CrystalExplorer (CE17/CE21), DFTB3-D3BJ, and PM7 against experimental stability data for 14 chlorine- and bromine-containing polymorphs across six CSD families. Chlorine systems show method-consistent performance, but bromine introduces large lattice energy variations (>10 kJ/mol) and, in several cases, qualitatively wrong stability rankings. Crucially, low mean absolute errors do not ensure correct thermodynamic ordering, and no semi-empirical method proves universally reliable for bromine. Only PBE-D3BJ achieves perfect experimental agreement. These results position bromine-containing crystals as exceptionally sensitive benchmarks and emphasize internal validation against experiment or reference DFT as essential before large-scale studies—particularly timely as machine-learning potentials emerge.

## 1. Introduction

The selection of an appropriate computational method is a fundamental challenge in modern chemical research, particularly for complex materials such as organic crystals [1,2,3,4,5]. The landscape of available techniques is vast, spanning from highly accurate but computationally expensive ab initio methods (e.g., Coupled Cluster) to more efficient approaches like Density Functional Theory (DFT) and further to semi-empirical and force-field-based methods designed for high-throughput screening [6,7,8,9]. For many routine tasks involving periodic systems, the choice inevitably involves a trade-off between accuracy and computational cost.

Ab initio methods, while prized for their predictive power, scale steeply with system size and are therefore typically limited to small molecules [10,11]. DFT has consequently become the workhorse of computational chemistry for the past three decades, offering a favorable balance of accuracy and efficiency across a broad range of problems [12,13]. However, DFT is not without its challenges. The accuracy of any calculation depends critically on the chosen exchange–correlation functional, and the lack of a systematic route to improvement means that performance is often system-dependent [14,15,16,17,18,19]. This has led to a proliferation of specialized functionals, which, although improving accuracy for targeted properties, complicate method selection for non-expert users and raise concerns about transferability [20,21,22,23,24].

To achieve even greater efficiency—for tasks such as crystal structure prediction (CSP) or screening large datasets—semi-empirical and tight-binding approaches remain indispensable [25,26,27,28,29,30]. Methods such as DFTB (Density Functional based Tight Binding) and parametrized semi-empirical models enable the study of systems that remain inaccessible to routine DFT [31,32,33,34,35]. Yet their reliability hinges critically on the quality and relevance of the underlying parameterization. Parameters are frequently derived from molecular datasets, and their transferability to periodic systems or to poorly represented elements cannot be taken for granted [36,37,38,39,40].

This issue is particularly acute for organic systems containing heavy halogens such as bromine. Bromine’s electronic structure, high polarizability, and propensity to form diverse non-covalent interactions (e.g., halogen bonding) present a stringent test for computational models [41]. If bromine parameters are poorly optimized, extrapolated from lighter halogens such as chlorine, or derived from limited molecular environments, calculations on bromine-containing crystals can become highly unreliable. At the same time, bromine-containing compounds are critically important in materials science, pharmaceuticals, and crystal engineering, making their accurate description a priority [42,43,44].

Thus, a one-size-fits-all strategy for method selection is untenable. For a new system, especially one containing an element such as bromine, rigorous internal benchmarking is a practical safeguard against misleading results. This entails testing a shortlist of candidate methods against a higher level of theory or, ideally, against experimental data for a representative subset of the systems under investigation. Only in this way can one assess whether a method’s known strengths and weaknesses are preserved for the specific chemical problem at hand.

In this work, we demonstrate the critical importance of such internal benchmarking using a set of bromine- and chlorine-containing organic crystal polymorphs. We evaluate the performance of several widely used semi-empirical approaches—CrystalExplorer (CE17, CE21), DFTB3, and PM7—against both periodic DFT (PBE-D3(BJ)) and experimental stability data. We show that while chlorine-containing structures are described with reasonable consistency, bromine-containing systems expose dramatic method-dependent variations in relative lattice energies. These results highlight bromine-containing crystals as exceptionally sensitive benchmarks and underscore the non-negotiable need for careful, system-specific method validation prior to any large-scale computational study.

## 2. Results

**Figure 1:**
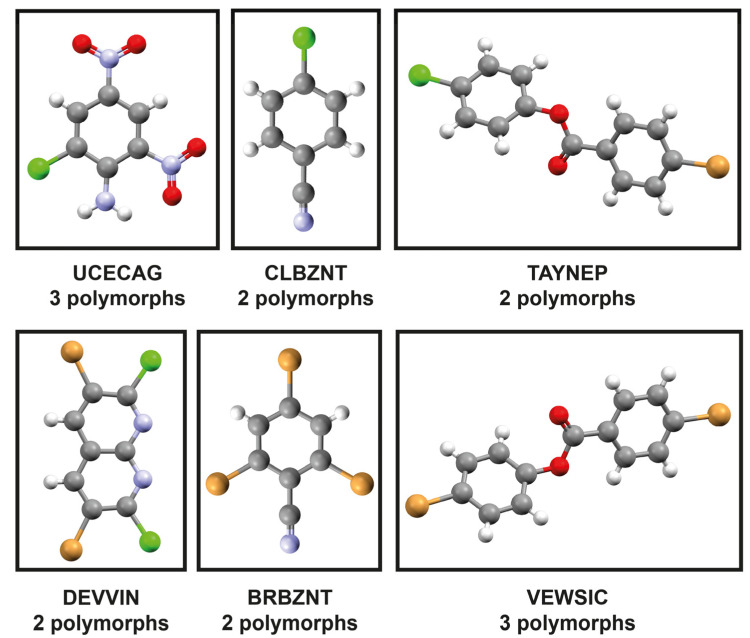
Examined crystal systems organized by polymorphic family: UCECAG (chlorine), CLBZNT (chlorine), TAYNEP (mixed chlorine/bromine), DEVVIN (mixed chlorine/bromine), BRBZNT (bromine), and VEWSIC (bromine). Atomic color scheme: carbon (gray), hydrogen (white), oxygen (red), chlorine (green), bromine (brown).

The study originated from discrepancies observed in semi-empirical lattice energies for mechanically responsive bromine-containing crystals, diverging sharply from periodic DFT references. This prompted the assembly of a limited but systematic benchmark set comprising 14 polymorphs across six carefully selected CSD families designed to probe halogen effects while maintaining high chemical similarity. All systems share a common structural motif of halogenated aromatic cores, limited heteroatom content (N, O, or ester linkages), and low molecular weight, allowing method performance to be attributed primarily to halogen type rather than to broader functional-group diversity. The benchmark includes two Cl-only families (UCECAG: 2-chloro-4,6-dinitroaniline; CLBZNT: 4-chlorobenzonitrile), two mixed Cl/Br families (TAYNEP: 4-chlorophenyl 4-bromobenzoate; DEVVIN: 3,6-dibromo-2,7-dichloro-1,8-naphthyridine), and two Br-only families (BRBZNT: 2,4,6-tribromobenzonitrile; VEWSIC: 4-bromophenyl 4-bromobenzoate) (Figure 1).

For each structure, lattice energies were evaluated using five computational approaches spanning a hierarchy of theoretical rigor and parameterization strategies: PBE-D3(BJ)—periodic DFT, serving as the reference method; CE17 and CE21—CrystalExplorer approach, parameterized specifically for organic crystals; DFTB3-D3(BJ)—a semi-empirical tight-binding method; PM7—a molecular semi-empirical method (MOPAC) developed and trained on molecular rather than periodic data. Two key metrics were examined: (i) the predicted stability order of polymorphs within each family, compared against experimental thermodynamic data if available, and (ii) the corresponding relative lattice energy (energy differences in kJ mol^−1^) (see Section 3.6 for details). The stability ranking tests a method’s ability to capture experimental preferences, while the numerical energy values reveal whether errors remain within acceptable bounds even when the ranking is correct (Table 1).

### 2.1. Chlorine-Containing Systems: A Baseline for Performance

For the chlorine-containing polymorphs (UCECAG and CLBZNT), all methods except CE17 show reasonable agreement with experiment and with the PBE reference. As shown in Table 1, PBE-D3(BJ) correctly reproduces the experimental stability order (where available) across all five structures (100% rank accuracy). CE21, DFTB3-D3(BJ), and PM7 achieve 60% rank accuracy, each making one error within the UCECAG family. CE17, however, fails dramatically: none of the five structures receives the correct stability rank, and only one of four polymorphic pairs (the CLBZNT pair) is ordered correctly—a performance statistically indistinguishable from random in this particular case (Table 2).

The mean absolute errors (MAEs) relative to PBE tell a similar story (Table 2). For individual structures, CE17, CE21, and DFTB3-D3(BJ) all perform well, with MAEs below 2.5 kJ mol^−1^. DFTB3-D3(BJ) is the most accurate among the approximate methods (MAE_str_ = 1.38 kJ mol^−1^; MAE_pair_ = 2.06 kJ mol^−1^). PM7, by contrast, exhibits substantially larger errors (MAE_str_ ≈ 10 kJ mol^−1^), indicating that a molecular parameterization transferred directly to periodic systems carries significant risk, even for relatively “well-behaved” chlorine-containing crystals.

Critically, these results also demonstrate that low MAE does not guarantee correct ranking. CE17 achieves a respectable MAE_str_ of 2.30 kJ mol^−1^ yet fails almost completely on stability order—a cautionary reminder that energetic accuracy and thermodynamic fidelity are not synonymous.

### 2.2. Mixed Chlorine/Bromine System: The First Signs of Instability

The TAYNEP and DEVVIN families, containing both chlorine and bromine, expose the first major divergence among methods. PBE-D3(BJ), CE17, DFTB3-D3(BJ), and PM7 all correctly identify TAYNEP01 as the more stable form (Table 1). CE21, however, predicts a complete inversion, assigning TAYNEP as the stable polymorph—an error of 9.4 kJ mol^−1^ in relative energy. Both CE17 and CE21 overestimate the stability of DIVVIN polymorph up to almost 6 and 9 kJ/mol (Table 1). This result indicates that bromine can be associated with larger method-dependent deviations than chlorine in the mixed systems studied here.

### 2.3. Bromine-Containing Systems: A Stress Test for Approximate Methods

The bromine-only families (BRBZNT and VEWSIC) reveal the full extent of the challenge. PBE-D3(BJ) again provides perfect agreement with experimental stability data (where available)—100% rank and pairwise accuracy. All approximate methods, however, show degraded performance (Table 1 and Table 2).

CE17, CE21, and PM7 each achieve 60% rank accuracy and 75% correct pairwise ordering, showing no decline from their performance on chlorine systems. More concerning is DFTB3-D3(BJ): while it maintains 60% rank accuracy, its pairwise accuracy plummets to 25%, meaning it systematically misorders the relative stability of polymorph pairs. This is not merely a matter of small numerical errors (as it was for chlorine systems); DFTB3-D3(BJ) inverts the stability of VEWSIC and VEWSIC01 by nearly 20 kJ mol^−1^ (Table 1), a qualitatively wrong prediction.

The energetic errors for bromine systems are correspondingly larger (Table 1 and Table 2). MAEs for individual structures rise sharply across all methods: CE17—from 2.30 kJ mol^−1^ (Cl) to 4.08 kJ mol^−1^ (Br); CE21—from 2.12 kJ mol^−1^ (Cl) to 7.42 kJ mol^−1^ (Br); DFTB3-D3(BJ)—from 1.38 kJ mol^−1^ (Cl) to 13.86 kJ mol^−1^ (Br); PM7—from 10.08 kJ mol^−1^ (Cl) to 39.22 kJ mol^−1^ (Br).

The extreme value for PM7 is driven primarily by VEWSIC02, a structure previously identified in the literature as crystallographically incorrect [42,51,52]. Excluding this outlier reduces PM7’s MAE_str_ for bromine to approximately 5.9 kJ mol^−1^ and halves the error for DFTB3-D3(BJ) to ~6 kJ mol^−1^, while for CE17 and CE21 it becomes 2.9 kJ mol^−1^ and 5.2 kJ mol^−1^ accordingly. This sensitivity to a single problematic structure suggests that, for the present limited benchmark, apparent differences in bromine performance may be influenced strongly by outlier effects.

### 2.4. Implications for Method Selection: Bromine as a Sensitive Benchmark

These results deliver three key messages. First, chlorine-containing organic crystals are described with reasonable consistency across computational methods, providing a baseline for comparison. Second, bromine systematically degrades semi-empirical method performance, with relative lattice energy errors often exceeding 10 kJ mol^−1^ and, critically, incorrect stability rankings in several cases. Third, no approximate method proves universally reliable for bromine systems—each exhibits characteristic weaknesses that chlorine benchmarks fail to reveal.

To quantify the relationship between energetic accuracy and thermodynamic fidelity, we observe only a partial correlation between mean absolute error (MAE) and ranking accuracy. For chlorine systems, methods with low MAE (<2.5 kJ mol^−1^) generally achieve acceptable rank performance (≥60%), yet CE17 illustrates a key disconnect: despite a moderate MAE of 2.30 kJ mol^−1^, it fails completely on stability ordering. This divergence intensifies for bromine, where DFTB3-D3BJ—initially the most accurate for chlorine (MAE 1.38 kJ mol^−1^)—shows sharply increased errors (MAE 13.86 kJ mol^−1^) and pairwise accuracy dropping to 25%. Such behavior arises because polymorph ranking depends on relative errors between closely spaced energy levels; modest average deviations can still invert orders when experimental gaps are small (~5 kJ mol^−1^), while uniformly large errors may preserve trends. Thus, MAE and rank accuracy represent complementary metrics requiring separate validation.

These observations align with benchmarking literature on halogen bonding, where approximate methods show system-dependent quantitative failures for heavy halogens [53,54]. The present work extends these findings from molecular dimers to periodic polymorphs, revealing that halogen bonding-related challenges in bromine crystals can lead to thermodynamically critical misrankings. Collectively, these results position bromine-containing crystals as exceptionally sensitive benchmarks and confirm that internal validation against experiment or reference DFT remains essential before applying approximate methods to large-scale studies of such systems.

## 3. Materials and Methods

### 3.1. Examined Crystal Structures

Experimental geometries were obtained from the Cambridge Structural Database (CSD 5.45, November 2024) for six polymorphic families: BRBZNT (2,4,6-tribromobenzonitrile, 2 forms), CLBZNT (4-chlorobenzonitrile, 2 forms), UCECAG (2-chloro-4,6-dinitroaniline, 3 forms), VEWSIC (4-bromophenyl 4-bromobenzoate, 3 forms), TAYNEP (4-chlorophenyl 4-bromobenzoate, 2 forms) and DEVVIN (3,6-dibromo-2,7-dichloro-1,8-naphthyridine, 2 forms). All approaches used these structures as input geometries for all calculations. Crystal format conversion was performed via CrystalShift software (https://github.com/shes73/CrystalShift, accessed on 13 April 2026) [55]. Experimental stability rankings derive from TGA/DSC and crystallization data from literature. Lattice energies in all computational methods were obtained from the difference between polymorphs directly (kJ/mol).

### 3.2. Periodic DFT (Reference)

Calculations were performed with VASP 5.4.4 [56,57,58,59] using the PBE [60] functional with D3(BJ) dispersion correction [61]. PAW [62,63] pseudopotentials with Monkhorst-Pack [64] k-point sampling were used, ensuring energy convergence below 2 meV atom^−1^. Cutoff energies and k-point meshes were: 500 eV with 3 × 2 × 4, 2 × 3 × 1 and 3 × 3 × 2 for UCECAG, UCECAG02 and UCECAG03; 550 eV with 8 × 2 × 4 and 4 × 3 × 1 for CLBZNT07 and CLBZNT16; 550 eV with 3 × 1 × 2 and 2 × 2 × 2 for BRBZNT01 and BRBZNT02; 550 eV with 1 × 5 × 1 and 1 × 4 × 3 for TAYNEP and TAYNEP01; 550 eV with 2 × 3 × 2 and 1 × 3 × 2 for DEVVIN and DEVVIN01 and 800 eV with 2 × 5 × 1, 3 × 4 × 1 and 6 × 2 × 1 for VEWSIC, VEWSIC01 and VEWSIC02. Structures were fully optimized.

### 3.3. CrystalExplorer (CE17/CE21)

Calculations were performed using CrystalExplorer 21 (and 17.5) [65,66,67], which employs crystal structures without any optimization except for default normalization of hydrogen atom positions. A molecular cluster radius of 32 Å was used for all forms, providing energy convergence tighter than 0.1 kJ/mol. Lattice energies were obtained from intermolecular interaction energies calculated with the CE-B3LYP/6-31G(d,p) model incorporated in CrystalExplorer 21 (and 17.5), using the Gaussian 09 [68] program suite as a backend and k_ele_, k_pol_, k_dis_, k_rep_ default coefficients.

### 3.4. DFTB3-D3(BJ)

DFTB+ 24.1 [69] was employed with the third-order DFTB3 method, D3(BJ) dispersion correction, and the 3ob-3-1 Slater–Koster parameter set [70]. Structures were fully optimized. SCF convergence was set to 10^−5^ a.u., with k-point sampling matching the DFT calculations. Cluster expansion on supercells, constructed in accordance with the k-point meshes used for periodic DFT, yielded the lattice energies. Maximum angular momentum, Hubbard Derivatives for each atomic species, as well as damping hydrogen correction, and DFTD3 (BJ) dispersion correction parameters were set as required by Third-Order Parametrization for Organic and Biological Systems (3OB) in accordance with ref. [34,70].

### 3.5. MOPAC PM7

MOPAC2016 [71] with the PM7 Hamiltonian [72] was used. Input files were prepared using the MAKPOL software (https://openmopac.net/Manual/makpol.html, accessed on 13 April 2026). Clusters of molecules were provided as supercells with the following dimensions: 1 × 1 × 2, 2 × 4 × 1 and 2 × 2 × 1 for UCECAG, UCECAG02 and UCECAG03; 3 × 1 × 2 and 3 × 2 × 1 for CLBZNT07 and CLBZNT16; 3 × 2 × 2 and 1 × 2 × 1 for BRBZNT01 and BRBZNT02; 1 × 2 × 2 and 1 × 3 × 1 for TAYNEP and TAYNEP01; 1 × 2 × 1 and 1 × 2 × 1 for DEVVIN and DEVVIN01; and 1 × 3 × 1, 2 × 2 × 1 and 3 × 1 × 1 for VEWSIC, VEWSIC01 and VEWSIC02. Structures were fully optimized. It performs periodic calculations only at the Gamma point of the Brillouin zone, which corresponds to simple periodic boundary conditions for the electronic orbitals.

### 3.6. Data Analysis

Relative lattice energies were obtained by setting the most stable form (experimental or PBE reference) as a reference (zero) for each family. Method performance was assessed via: rank accuracy—percentage of structures with correct experimental stability order; pairwise accuracy—percentage of polymorph pairs correctly ordered; mean absolute errors (MAEs) relative to PBE for individual structures (MAE_str) and polymorph pairs relative energies (MAE_pair). Bootstrap resampling (1000 iterations) provided 95% confidence intervals for MAEs.

## 4. Conclusions

This study demonstrates that bromine-containing organic crystals pose a substantial challenge for a wide range of computational methods, including those commonly considered reliable for organic systems. While chlorine-containing structures exhibit consistent energetic trends across all examined approaches, the introduction of bromine leads to dramatic method-dependent variations in relative lattice energies and, in some cases, qualitatively incorrect stability rankings.

Among the methods tested, only periodic DFT (PBE-D3(BJ)) consistently reproduced experimental stability orders across all halogen-containing families. Semi-empirical and tight-binding approaches showed variable performance: CrystalExplorer21 and DFTB3-D3(BJ) delivered reasonable accuracy for chlorine systems but degraded significantly for bromine, while PM7 proved particularly sensitive to outlier structures such as VEWSIC02.

Crucially, low mean absolute errors did not guarantee correct thermodynamic ranking, underscoring that energetic accuracy and stability ordering are distinct metrics requiring separate validation. The reverse is equally true: correct ranking does not imply that a method’s energies are quantitatively reliable for specific property calculations.

These findings lead to a clear and practical conclusion: internal benchmarking is not merely a recommended precaution but an essential prerequisite for any computational study, especially involving bromine-containing organic crystals. Reliance on a single approximate method, chosen without validation against experiment or a higher level of theory, carries a substantial risk of producing misleading thermodynamic data.

More broadly, bromine-containing crystals in this dataset provide a sensitive benchmark for method assessment. Their complex electronic structure and diverse non-covalent interactions provide a rigorous test for accuracy, transferability, and robustness. As the field moves toward next-generation approaches—including machine-learning potentials—continued attention to these challenging systems will be critical for ensuring that new methods deliver genuine predictive power rather than merely reproducing the limitations of their training data.

## Figures and Tables

**Table 1:** Relative lattice energies (kJ mol^−1^) and stability ranks (Roman numerals) for chlorine- and bromine-containing crystal structures.

Hal	Refcode	Stability *	PBE- D3(BJ)	CE17	CE21	DFTB3- D3(BJ)	MOPAC PM7
Cl	UCECAG03	I	0 (I)	0.3 (II)	0 (I)	0 (I)	0 (I)
	UCECAG	II	0.7 (II)	1.1 (III)	5.8 (II)	2.1 (II)	18.9 (II)
	UCECAG02	III	9.2 (III)	0 (I)	6.2 (III)	11.3 (III)	32.9 (III)
	CLBZNT16	I **	0 (I)	1.5 (II)	2.1 (II)	3.0 (II)	8.1 (II)
	CLBZNT07	II **	0.4 (II)	0 (I)	0 (I)	0 (I)	0 (I)
Cl-Br	TAYNEP01	I	0 (I)	0 (I)	9.4 (II)	0 (I)	0 (I)
	TAYNEP	II	4.8 (II)	5.7 (II)	0 (I)	7.7 (II)	16.9 (II)
	DIVVIN01	I **	0 (I)	5.1 (II)	8.4 (II)	0 (I)	0 (I)
	DIVVIN	II **	0.6 (II)	0 (I)	0 (I)	14.4 (II)	0.1 (II)
Br	BRBZNT02	I	0 (I)	0.5 (II)	0 (I)	0 (I)	3.2 (II)
	BRBZNT01	II	4.7 (II)	0 (I)	3.2 (II)	3.1 (II)	0 (I)
	VEWSIC	II **	4.7 (II)	10.9 (II)	23.9 (III)	0.7 (II)	20.0 (II)
	VEWSIC01	I **	0 (I)	0 (I)	0 (I)	19.9 (III)	0 (I)
	VEWSIC02 ***	III	23.9 (III)	14.9 (III)	7.5 (II)	0 (I)	196.8 (III)

* Experimental stability order, derived from thermal analysis (TGA, DSC), crystallization experiments, and other data reported alongside the crystallographic information available in the Cambridge Structural Database (CSD) [42,45,46,47,48,49,50]. ** In the absence of quantitative experimental data [42,50] in the case of VEWSIC, DIVVIN, and equal 1.2 kJ/mol enthalpies of CLBZNT07/16 phase transitions to form III, the stability ranking is based on DFT (PBE) calculations performed in this study. *** Reported crystal structure has been identified as incorrect in the literature [42,51,52]; 4-bromophenyl 4-bromobenzoate does not adopt this structure in its crystalline form, and it is therefore expected to be energetically less favorable.

**Table 2:** Accuracy of predicted stability ranks (%) and mean absolute errors (kJ mol^−1^) relative to PBE-D3(BJ) for halogen-containing crystal families (structures/pairs) *.

Approach	Cl (5/4)	Cl-Br (4/2)	Br (5/4)
Accuracy of predicted stability ranks for structures/pairs (%)			
PBE-D3(BJ)	100/100	100/100	100/100
CE17	0/25	50/50	60/75
CE21	60/75	0/0	60/75
DFTB3-D3(BJ)	60/75	100/100	60/25
PM7 MOPAC	60/75	100/100	60/75
**Mean absolute errors (kJ mol^−1^) relative to PBE-D3(BJ) presented as MAE_str_/MAE_pair_**			
CE17	2.30/4.98 [1.27–3.68]/[2.34–8.45]	1.65/3.3 [0.0–5.7]/[0.9–5.7]	4.08/4.96 [1.41–6.71]/[2.11–8.64]
CE21	2.12/3.78 [0.94–3.74]/[1.89–6.02]	5.80/11.6 [0.0–14.2]/[9.0–14.2]	7.42/10.6 [2.81–13.9]/[5.12–17.3]
DFTB3-D3(BJ)	1.38/2.06 [0.89–3.78]/[0.41–2.60]	4.18/8.4 [0.0–13.8]/[2.9–13.8]	13.86/11.5 [4.67–20.1]/[4.67–20.1]
PM7 MOPAC	10.08/8.26 [3.45–14.9]/[3.45–14.9]	3.15/6.3 [0.0–12.1]/[0.5–12.1]	39.22/33.8 [8.92–68.4]/[8.92–68.4]

* Stability metrics are reported as rank accuracy (%: correct structure rankings per family)/pairwise accuracy (%: correctly ordered polymorph pairs). Chlorine (Cl: 5 structures/4 pairs across UCECAG, CLBZNT families); mixed Cl/Br (4 structures/2 pairs, TAYNEP, DIVVIN families); bromine (Br: 5 structures/4 pairs across BRBZNT, VEWSIC families). MAEs of relative lattice energies are given as MAE_str_ (individual structures)/MAE_pair_ (polymorph pairs), with 95% confidence intervals from 1000 bootstrap iterations in brackets (see Section 3.6). CLBZNT, DEVVIN, VEWSIC family’s stability is partly based on PBE calculations due to limited experimental data; VEWSIC02 is noted as an incorrectly solved structure from the literature.

## Data Availability

The data presented in this study are available on reasonable request from the corresponding author.

## Abbreviations

The following abbreviations are used in this manuscript:

DFT	Density Functional Theory
PBE-D3(BJ)	Perdew–Burke–Ernzerhof functional with Grimme’s dispersion correction (D3) and Becke–Johnson damping
CE17	CrystalExplorer 17
CE21	CrystalExplorer 21
DFTB3-D3(BJ)	Density Functional Tight Binding, third order, with D3(BJ) dispersion correction
CSP	Crystal Structure Prediction
DFTB	Density Functional based Tight Binding
CSD	Cambridge Structural Database
TGA	Thermogravimetric Analysis
DSC	Differential Scanning Calorimetry
MAE	Mean Absolute Error
MAE_str_	Mean Absolute Error for individual structures
MAE_pair_	Mean Absolute Error for polymorph pairs
VASP	Vienna Ab initio Simulation Package
3ob-3-1	Third-order DFTB parameter set for organic and biological systems
SCF	Self-Consistent Field
